# Increasing the robustness of *Escherichia coli* for aromatic chemicals production through transcription factor engineering

**DOI:** 10.1007/s44307-024-00023-x

**Published:** 2024-04-02

**Authors:** Xiao-Ling Zhou, Meng-Sang Zhang, Xing-Run Zheng, Zhi-Qian Zhang, Jian-Zhong Liu

**Affiliations:** 1 State Key Laboratory of Biocontrol, School of Life Sciences, Sun Yat-Sen University, Guangzhou, 510275 People’s Republic of China; 2 Joint Research Center of Engineering Biologylogy Technology of Sun Yat-Sen University and Tidetron Bioworks, Guangzhou, 510275 China; 3 Tidetron Bioworks Technology (Guangzhou) Co., Ltd. Guangzhou, Guangzhou, 510399 China

**Keywords:** Robustness, Aromatic chemicals, Transcription factor, *Escherichia coli*

## Abstract

Engineering microbial cell factories has been widely used to produce a variety of chemicals, including natural products, biofuels, and bulk chemicals. However, poor robustness limits microbial production on an industrial scale. Microbial robustness is essential to ensure reliable and sustainable production of targeted chemicals. In this study, we developed an approach to screen transcription factors to improve robustness using CRSPRa technology. We applied this approach to identify some transcription factors to increase the robustness of *Escherichia coli* to aromatic chemicals. Activation of *hdfR, yldP, purR, sosS, ygeH, cueR, cra*, and *treR* increased the robustness of *E. coli* to phenyllactic acid. Upregulation of some transcription factors also improved the robustness to caffeic acid (*cra*) or tyrosol (*cra, cueR, treR, soxS, hdfR* and *purR*). Our study demonstrated that transcription factor engineering using CRISPRa is a powerful method to increase microbial robustness. This research provides new approaches to efficiently find genes responsible for increasing microbial robustness.

**Supplementary Information:**

The online version contains supplementary material available at 10.1007/s44307-024-00023-x.

## Introduction

With the advances in synthetic biology and metabolic engineering, engineering microbial cell factories has been widely applied to produce various chemicals, such as natural products, biofuels, and bulk chemicals. Engineering microbial cell factories constantly face disturbances resulting from metabolic imbalance, genetic and phenotypic instability, and various harsh industrial conditions (including low pH, high temperature, and metabolite toxicity, etc.) in the process of large-scale fermentation, resulting in poorly performing strains under industrial conditions. However, the design of microbial cell factories in the laboratory often does not take into account these multiple perturbations encountered in industrial conditions. Increasing the strain robustness against these conditions is therefore one of the most important considerations in engineering microbial cell factories. Microbial robustness refers to the ability of the microbe to maintain constant production performance (defined as titers, yields, and productivity) regardless of the various stochastic and unpredictable perturbations that occur during bioprocessing.

Transcription factors are key proteins controlling the expression of ‘target genes’. Cells have evolved to optimize cellular function through the coordinated regulation of multiple enzymes and pathways by different transcription factors in response to different environmental conditions. Transcription factor engineering is emerging as a viable and efficient approach to improve strain robustness (Mohedano et al. 2022; Tao et al. 2022; Gong et al. 2017; Xu et al. 2024). The global transcription machinery engineering of σ^70^ (Alper and Stephanopoulos 2007) and global transcription factor cAMP receptor protein (CRP) (Zhang et al. 2012; Chong et al. 2014) successfully improved solvent tolerance, acid tolerance, and osmotolerance. Overexpression of the response regulator DR1558 from *Deinococcus radiodurans* in *E. coli* increased tolerance to osmotic stress at high concentrations of 300 g/L glucose and 2 mol/L NaCl (Guo et al. 2017). Heterologous expression of the global regulator *irrE* from *D. radiodurans* and its mutant increased tolerance to ethanol or butanol stress in *E. coli* by 10 to 100 fold (Chen et al. 2011). The regulon-specific transcription factor Hass1 was engineered to improve acetic acid tolerance in *Saccharomyces cerevisiae* (Swinnen et al. 2017). Overexpressing GlxR, RamA and SugR significantly improved the N-acetylglucosamine biosynthesis in *C. glutamicum* (Deng et al. 2021)*.* These results demonstrate that engineering transcription factors can improve microbial resistance. However, can engineering transcription factors increase strain robustness or not? Although the terms robustness and tolerance are sometimes used interchangeably in industrial microbial applications, they refer to different concepts. Tolerance (or resistance) denotes the ability of cell growth when exposed to a single or multiple perturbations. It is generally only described by growth-related parameters (such as viability or specific growth rate). Robustness represents the ability of a strain to maintain a stable production performance (quantified as titer, yield, and productivity) when conditions change. There is clear evidence that tolerance improvements can increase production. However, improvements in tolerance are not sufficient to guarantee an increase in chemical production, i.e. robustness.

There are 172 transcription factors in the *E. coli* genome. Can these transcription factors be engineered to improve robustness? Recently, Clustered Regularly Interspaced Short Palindromic Repeats activation (CRISPRa) using SoxS as an activator has been developed for transcriptional upregulation (Dong et al. 2018; Fontana et al. 2020). The SoxS-CRISPRa system contains a scaffold RNA (ScRNA), which is a modified version of gRNA containing an MS2 RNA stem-loop at its 3’ end (Fig. S1). This ScRNA interacts with the corresponding MS2 coat protein (MCP) fused to the transcriptional activator SoxS, which recruits the RNAP holoenzyme to a promoter of choice. We have used the SoxS-CRISPRa system to identify genes that should be activated for the production of pinene (Niu et al. 2019), astaxanthin (Lu et al. 2022) and 4-hydroxyphenylacetic acid (Shen et al. 2022).

Thus, we applied the SoxS-CRISPRa system to engineer *E. coli* endogenous transcription factors to improve robustness. All *E. coli* endogenous transcription factor genes were activated by CRISPRa to identify the target genes for increasing robustness to aromatic chemicals.

## Materials and methods

### Strains, plasmids and primers

The bacterial strains and plasmids used in this study are listed in Table 1. The primers used in this study are listed in Supplementary Table 1.

**Table 1:** Strains and plasmids used in this study

Name	Description	Sources
Strain		
* E. coli* DH5α	F- *endA*1 *glnV*44 *thi*-1 *recA*1 *relA*1 *gyrA*96 *deoR nupG* Φ80 *dlacZ* Δ*M15* Δ(*lacZYA-argF*) U169	Invitrogen
PHE02	L-Phenylalanine producer, NST74, P_tyrA_::Esa-P_easS_, ΔdinB::P37-aroG^fbr^-pheA^fbr^	(Liu and Zhou 2022)
PHE02 (pZBK-P_esaR_-Cn*ldhA*)	Phenyllactic acid producer	(Liu and Zhou 2022)
* E. coli* TYR-14B1	L-tyrosine producer obtained from the combined ARTP mutagenesis and ep-WGS of *E. coli* TYR-30	(Yan et al. 2021)
* E. coli* TYR-14B1(pZEA-RgTAL-PaHpaB-SeHpaC)	Caffeic acid producing strain	lab storage
* E. coli* TYR-14B1(pZEA(U)-SpyTag-aro10-linker-yahK)	Tyrosol producer	lab storage
Plasmid		
pZBK-P_esaS_IC-P_esaR_AS	Quorum sensing plasmid, pBBR1 *ori*, P37 promoter, kan^r^	(Shen et al. 2019)
pZBK-P_esaR_-Cn*ldhA*	pZBK-P_esaS_IC-P_esaR_AS contain lactate dehydrogenase genes (GeneBank: AM260479) from *Cupriavidus necator* H16 under the control of the Esa-P_esaR_ activation system	(Liu and Zhou 2022)
pZBK	BglBrick/ePathBrick expression vector, pBBR1 *ori*, P37 promoter, kan^r^	(Li et al. 2015)
pZEA-RgTAL-PaHpaB-SeHpaC	pZBK containing the caffeic acid biosynthesis pathway genes	Our lab storage
pZEA(U)-SpyTag-aro10-linker-yahK	pZBK containing the tyrosol biosynthesis genes	Our lab storage
pBbB2K-dCas9*-MCPSoxS	CRIPRa plasmid, pBbB2K-dCas9* containing the MCPSoxS sequences	(Niu et al. 2019)
pTargetA	*E. coli* scRNA expression vector, BglBrick vector, P_tet_ promoter, Spe^r^, pMB1 *ori*	(Niu et al. 2019)
pZEABP	Constitutive expression vector, pBR322 *ori*, P37 promoter, Amp^r^	(Li et al. 2015)

### CRISPRa of transcription factor genes

CRISPR activation of the transcription factor gene was performed as described by Niu et al. (2019). The N20 sequence was designed to target 60–100 bases upstream of the transcription start site, used to construct ScRNA plasmid. The ScRNA plasmids pTargetA-X series were obtained by reverse PCR from pTargetA, then cleaved with *Kpn*I and self-ligated.

CRISPRa plasmid pBbB2K-dCas9*-MCPSoxS and ScRNA vector pTargetA-X were co-transferred into the phenyllactic acid-producing strain *E. coli* (pZBK-P_esaR_-Cn*ldhA*). A single colony was inoculated into a 48-well microplate (4.6 mL) containing 1 mL LB medium, and then cultured at 37°C and 1000 rpm for 24 h on a shaker (MBR-420FL, TAITEC, Japan). After 2 h, 200 nM dehydrated tetracycline and 20 g/L phenyllactic acid were added sequentially to each 48-well microplate.

### Replacement of promoter

Replacement of the promoter with the P37 promoter was performed using the CRISPR-Cas method as previously described (Lu et al. 2022; Jiang et al. 2015; Shen et al. 2021). The replacement sgRNA plasmid pTargetB-X was prepared as described above for the CRISPRa system. The upstream and downstream homology arms of the native promoter were amplified and then sequentially cloned into the *Mlu*I/*Avr*II and *Nhe*I/*Kpn*I of pZBK. The targeting fragment was excised from the above plasmid using *Mlu*I/*Kpn*I and then transferred into the electrocompetent cells harboring pCas* and pTargetB-X to replace the corresponding gene.

### Production of aromatic compound

Fermentations were carried out in 250 mL Erlenmeyer flasks containing 50 mL fermentation medium. The seed liquid was inoculated into the fermentation medium with a starting OD_600_ of 0.1, and the fermentation cultures were shaken at 37°C for the production of phenyllactic acid and caffeic acid or 30°C for the production of tyrosol, 200 rpm for 72 h. The fermentation medium for the production of phenyllactic acid (M8) contained 9 g/L KH_2_PO_4_, 18 g/L Na_2_HPO_4_, 0.58 g/L NH_4_Cl, 4 g/L yeast extract, 0.5 g/L NaCl, 0.61 g/L MgSO_4_·7H_2_O, 0.008 g/L CaCl_2_, 28.05 g/L glucose, 50 mg/L thiamine HCl and 2 ml/L of trace element I. The trace element solution I contained 2 g/L ZnSO_4_·7H_2_O, 1.1 g/L H_3_BO_3_, 0.5 g/L MnCl_2_·4H_2_O, 0.5 g/L FeSO_4_·7H_2_O, 0.16 g/L CoCl_2_·6H_2_O, 0.16 g/L CuSO_4_·5H_2_O, 0.11 g/L (NH_4_)_6_Mo_7_O_24_·4H_2_O, 5 g/L EDTA. The fermentation medium for the production of caffeic acid and tyrosol (M9GT) contained 6.78 g/L Na_2_HPO_4_, 3 g/L KH_2_PO_4_, 1 g/L NH_4_Cl, 2.5 g/L yeast extract, 0.5 g/L NaCl, 120.4 mg/L MgSO_4_, 11.1 mg/L CaCl_2_, 20 g/L glucose and trace elements II. The final concentrations of trace elements II were 100 mg/L FeSO_4_⋅7H_2_O, 13.5 mg/L CaCl_2_, 22.0 mg/L ZnSO_4_⋅7H_2_O, 5.8 mg/L MnSO_4_⋅4H_2_O, 10.0 mg/L CuSO_4_⋅5H_2_O, 1.0 mg/L (NH_4_)_6_Mo_7_O_24_⋅4H_2_O, 2.0 mg/L Na_2_B_4_O_7_⋅10H_2_O, and 0.1 ml of 35% (w/w) HCl per litre. Ampicillin, kanamycin was added to the media as required at final concentrations of 100 and 50 mg/mL, respectively.

### Quantitative real-time PCR (qRT-PCR)

*E. coli* cells grown in a fermentation medium for 36 h were used to extract RNA using an RNA pure kit (Magen, Beijing, China). The cDNA was synthesized with a FastKing RT kit (TIANGEN, Beijing, China). Quantitative real-time PCR was performed with Talent qPCR PreMix (TIANGEN, Beijing, China), and reaction mixtures were subjected to qPCR in a StepOne thermocycler (Applied Biosystems, USA). The PCR program was as follows: 95 °C for 10 min, followed by 45 cycles of denaturation at 95 °C for 10 s, annealing at 60 °C for 20 s, and extension at 72 °C for 15 s. Expression levels were analyzed using the 2^−△△Ct^ method described by Livak and Schmittgen (2001) and normalized using *cysG* gene expression as a reference.

### Cell growth and chemical quantification assay

Growth was determined by measuring OD at 600 nm. Aromatic compounds in the supernatants were analyzed by HPLC (LC-20A, Shimadzu, Japan) with an Inertsil ODS-SP column (5 μm, 4.6 × 150 mm, GL Sciences Inc., Tokyo, Japan). For phenyllactic acid analysis, the flow rate was 1.0 mL/min at 37°C. The mobile phase consisted of H_2_O (buffer A, supplemented with 0.05% trifluoroacetic acid) and methanol (buffer B, supplemented with 0.05% trifluoroacetic acid). The gradient program was as follows: 10 to 60% buffer B for 8 min, 60% buffer B for an additional 15 min, and 60 to 10% buffer B for 20 min. For caffeic acid and tyrosol analysis, methanol containing 0.2% TFA was used as the mobile phase. For caffeic acid analysis, the flow rate was 1.0 mL/min at 30°C. The methanol concentration was increased from 10 to 90% for 20 min, and then decreased to 10% for 5 min. For tyrosol analysis, the methanol concentration was increased from 14 to 45% for 20 min, and then decreased to 14% and maintained at this concentration for 10 min at a flow rate of 0.5 mL/min at 30°C. All chemicals were quantified based on their specific wavelength and peak area (210 nm for phenyllactic acid, 280 nm for tyrosol, and 321 nm for caffeic acid).

## Results and discussion

### Screening transcription factors to increase the robustness to phenyllactic acid

**Fig. 1:**
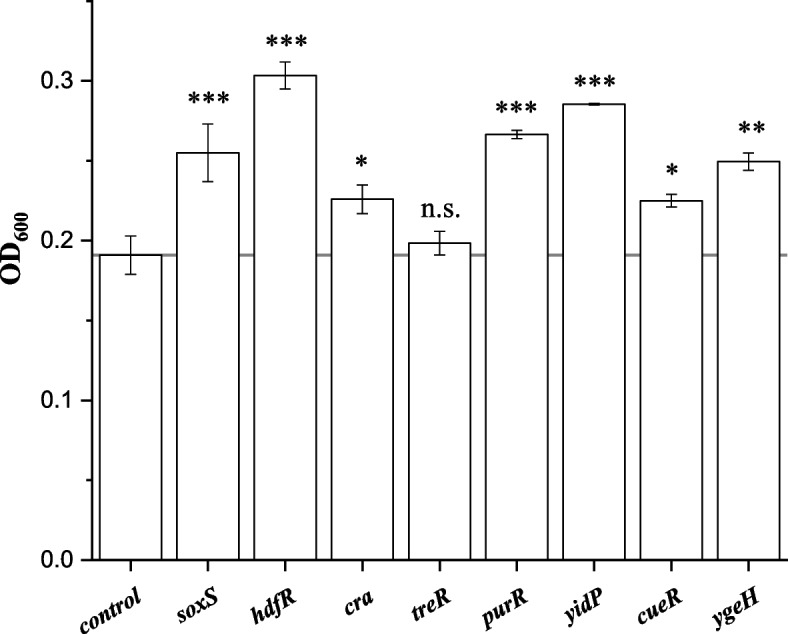
Effect of CRISPRa of the selected transcription factors on the growth of phenyllactic acid producing *E. coli* PHE02 (pZBK-P_esaR_-Cn*ldhA*) in the presence of 15 g/L phenyllactic acid in shake flasks. Significance was performed by two-tailed *t*-test: not significant (n.s.), *p* ≤ 0.05 (*), *p* ≤ 0.01 (**), and *p* ≤ 0.001 (***)

Microbial robustness denotes the ability of a microorganism to maintain a stable production performance (titer, productivity and yield) when exposed to varying conditions. Tolerance (or resistance) denotes the ability of cell growth when conditions change. Because the robustness strain must have a higher tolerance, we first screened transcription factors to increase the resistance to phenyllactic acid, and then investigated the effects of these screened transcription factors on the production of phenyllactic acid. There are 172 transcription factors in the *E. coli*. We first investigate the activation effects of the 172 transcription factors on the resistance of the phenyllactic acid-producing *E. coli* to phenyllactic acid. We constructed scRNA-expressing plasmids of these transcription factors and co-transferred with the CRIPRa plasmid into the phenyllactic acid producing-*E. coli* PHE02 (pZBK-P_esaR_-Cn*ldhA*) for microplate analysis of growth. We first determined the effect of phenyllactic acid on the growth of *E. coli* PHE02. 15 g/L or 20 g/L phenyllactic acid showed significant inhibition on the growth (Fig. S2). Thus, 20 g/L phenyllactic acid was selected in the screening experiments. As shown in Fig. S3, activation of some transcription factors improved cell growth in the presence of 20 g/L phenyllactic acid and increased the resistance to phenyllactic acid. The strains with higher cell growth in the microplate were selected for further shake flask analysis. Because the OD600 values at 20 g/L phenyllactic acid in the shake flask culture were too low, we also determined the growth at 15 g/L phenyllactic acid in the shake flask culture and the data of the tolerant genes to are presented in Fig. 1. As shown in Fig. 1, activation of *hdfR, yidP, purR, sosS, ygeH, cueR,* and *cra* increased the resistance to phenyllactic acid. Tre showed insignificantly effect on growth.

Then, we investigated the effects of CRISPR activation of the selected transcription factors on the production of phenyllactic acid. As shown in Table 2, the activations of the 8 transcription factors improved the production of phenyllactic acid by 5.2–76.2%. Thus, the activations of these transcription factors increased the resistance to phenyllactic acid and the production of phenyllactic acid, indicating the increased robustness to phenyllactic acid. The *soxS* gene encodes the regulator protein SoxS.It activates transcription of the genes of the SoxRS regulon, which provides cell’s antioxidant defense. The activation of the oxidative stress-related genes (*soxS*) increases the level of reactive oxygen species (ROS) in vivo, resulting in cell damage. To protect the cells from oxidative damage, the strain should strengthen the antioxidant defense system. Phenyllactic acid and other aromatic compounds show higher antioxidative activity. It indicates that the upregulation of the oxidative stress-related genes induced to synthesize more antioxidant (phenyllactiv acid) to protect the cells from oxidative damage. Lu et al. (2019) applied the strategy of oxidative stress eengineering to improve astaxanthin production in *E. coli*. The similar strategy was also successfully used to improve strain tolerance and production (Xu et al. 2018; Qin et al. 2020; Zhu et al. 2019). Global transcriptional regulator Cra (synonyms *fruR*) is a catabolite repressor/activator. It activates tricarboxylic acid (TCA) cycle and gluconeogenic enzyme (such as PpsA, Fbp, PckA and AceA), and represses the glycolytic enzymes, such as FruB, PfkA, PykF and AdhE. It was reported that overexpressing *ppsA* increased the aromatic compound production with near theoretic yield (Patnaik and Liao 1994). HTH-type transcriptional regulator HdfR negatively regulates the transcription of the flagellar master operon flhDC by binding the upstream region of the operon. Transcriptional regulator TreR is known to be responsive to trehalose. HTH-type transcriptional regulator PurR represses transcription of the genes involved in the de novo synthesis of purine nucleotides, regulating *purB*, *purC*, *purEK*, *purF*, *purHD*, *purL*, *purMN* and *guaBA* expression. In addition, it participates in the regulation or coregulation of genes involved in de novo pyrimidine nucleotide biosynthesis, salvage and uptake (*pyrC*, *pyrD*, *carAB* and *codBA*), and of several genes encoding enzymes necessary for nucleotide and polyamine biosynthesis (*prsA*, *glyA*, *gcvTHP*, *speA*, and *glnB*). These biosynthetic pathways of nucleotides involve many ATP-consuming enzymes. The upregulation of *purR* may provide more ATP for the production of phenyllactic acid. HTH-type transcriptional regulator CueR regulates the transcription of the *copA* and *cueO* genes. It activates transcription in response to increasing copper concentrations.

**Table 2:** Effects of CRISPR activation of the selected transcription factors on the production of phenyllactic acid in *E. coli* PHE02 (pZBK-P_esaR_-Cn*ldhA*)

Transcription factor	OD_600_	Phenyllactic acid (g/L)
SoxS	7.49 ± 0.06	2.27 ± 0.03*
HdfR	7.73 ± 0.03	2.39 ± 0.02*
Cra	13.19 ± 0.85	3.77 ± 0.28***
TreR	10.19 ± 0.96	2.90 ± 0.22**
PurR	13.80 ± 1.53	3.58 ± 0.30**
YidP	14.20 ± 0.73	3.96 ± 0.01***
CueR	13.75 ± 0.37	4.00 ± 0.01***
YgeH	11.95 ± 0.50	3.52 ± 0.39***
Control	5.72 ± 0.03	1.85 ± 0.02

Significance was performed by two-tailed *t*-test: not significant (n.s.), *p* ≤ 0.05 (*), *p* ≤ 0.01 (**), and *p* ≤ 0.001 (***)

To avoid the metabolic burden caused by the CRISPRa- and scRNA-expressing vectors, the native promoter of these transcription factors was replaced with the strong promoter P37 to upregulate them. Because YldP and YgeH are uncharacterized proteins, only six of the eight transcription factors were selected for promoter replacement. As shown in Table 3, the replacement of the native promoter of *treR, soxS* and *cueR* with the P37 promoter significantly increased the titer of phenyllactic acid by 8.9–10.5% and yield by 8.8–10.5%. Replacement of the native promoter of *cra* did not significantly improve the production of phenyllactic acid. Replacement of the native promoter of *purR* and *hdfR* significantly decreased the titer and yield of phenyllactic acid. TreR regulates the *treBC* operon involved in trehalose biosynthesis. We investigated the effect of the addition of trehalose in the fermentation medium on the production of phenyllactic acid. Adding 0.1–10 g/L trehalose to the medium significantly enhances the production of phenyllactic acid in the strain with the *treR* promoter replacement (Supplementary Table 2). Overexpression of the trehalose biosynthetic genes (*otsA*, *otsB*, and *treS*) from *Arthrobacter simplex* in *E. coli* can induce the cell to synthesize trehalose as an osmo-protector, thereby greatly improving its tolerance to ethanol (Cheng et al. 2020). The activity of the copper efflux regulator CueR is dependent upon the binding of copper. We investigated the effect of the addition of CuSO_4_ in the fermentation medium on the production of phenyllactic acid. The addition of 5 mM CuSO_4_ indeed increased the production of phenyllactic acid (Supplementary Table 3).

**Table 3:** Effects of the replacement of the promoter of the selected transcription factors on the production of phenyllactic acid

Host strain	Plasmid	OD_600_	Phenyllactic acid (g/L)	Yield (mg/g)
PHE02	pZBK-P_esaR_-Cn*ldhA*	9.81 ± 0.29	4.95 ± 0.05	106.1 ± 1.1
PHE02(P_cra_::P37)		9.79 ± 0.11	4.93 ± 0.13^n.s^	105.7 ± 2.8
PHE02(P_purR_::P37)		4.69 ± 0.29	1.81 ± 0.03***	54.8 ± 0.1
PHE02(P_hdfR_::P37)		5.25 ± 0.09	2.16 ± 0.08***	69.2 ± 1.9
PHE02(P_treR_::P37)		9.28 ± 0.01	5.39 ± 0.03*	115.4 ± 0.8
PHE02(P_soxS_::P37)		9.03 ± 0.34	5.47 ± 0.09**	117.2 ± 1.9
PHE02(P_cueR_::P37)		9.40 ± 0.06	5.43 ± 0.03*	116.4 ± 1.7

Significance was performed by two-tailed *t*-test: not significant (n.s.), *p* ≤ 0.05 (*), *p* ≤ 0.01 (**), and *p* ≤ 0.001 (***)

**Fig. 2:**
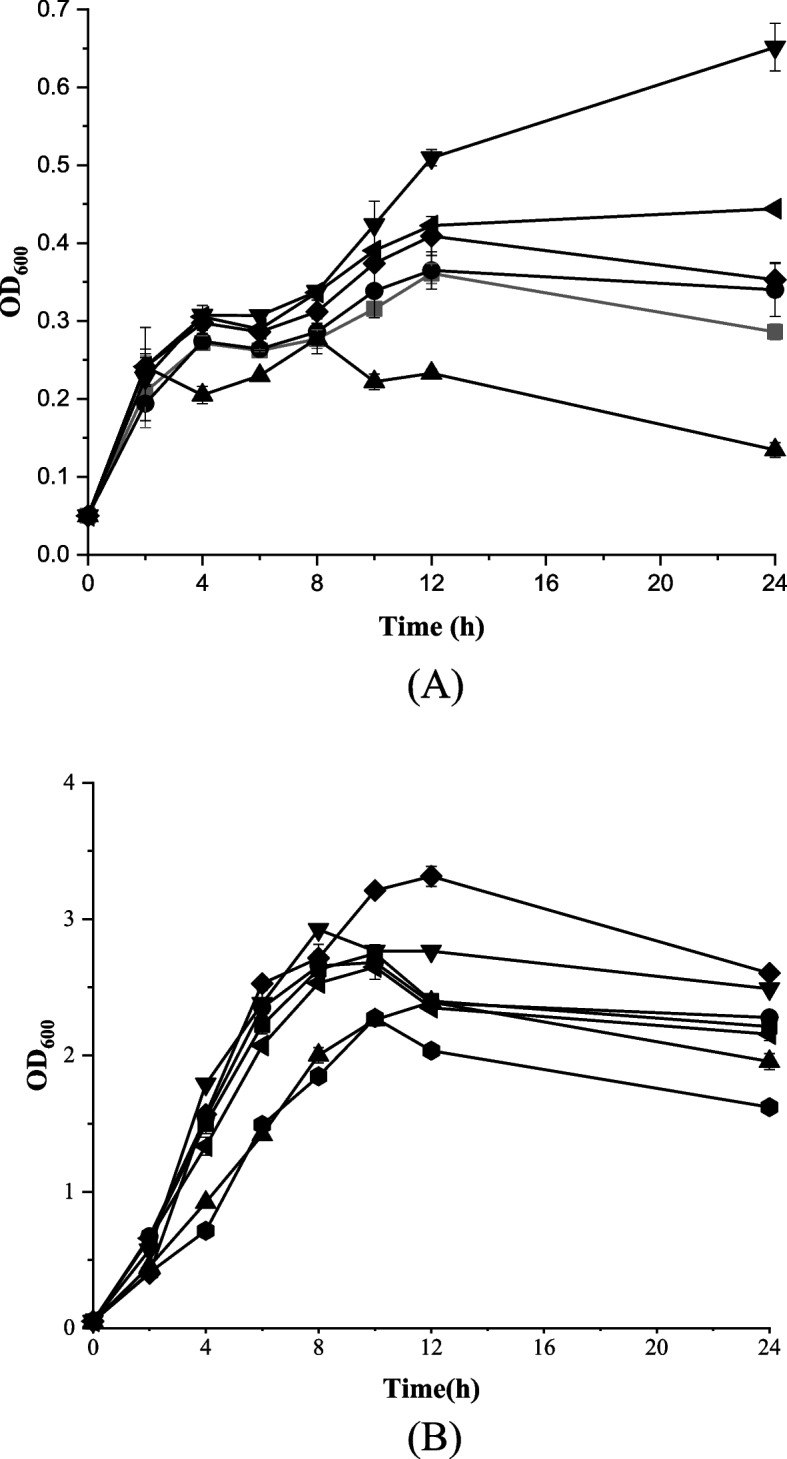
Effects of the replacement of the promoter of the selected transcription factors on the resistance with (**A**) and without (**B**) phenyllactic acid. (●)PHE02; (■) *craR*; (▲) *hdfR*; (▼) *treR*; (♦) *soxS*; (◄) *cueR;* *purR*

We also characterized the resistance of these strains to phenyllactic acid. As shown in Fig. 2A, the resistance to phenyllactic acid was improved after replacing the native promoters of the transcription factors of *treR*, *cueR* and *soxS.*The replacement of the *hdfR* promoter resulted in decreased tolerance. At the absence of phenyllactic acid, the replacement of the *soxS* and *treR* promoters improved growth (Fig. 2B). The promoter replacing strains of *creR* and *cueR* showed similar growth to the control strain.

**Fig. 3:**
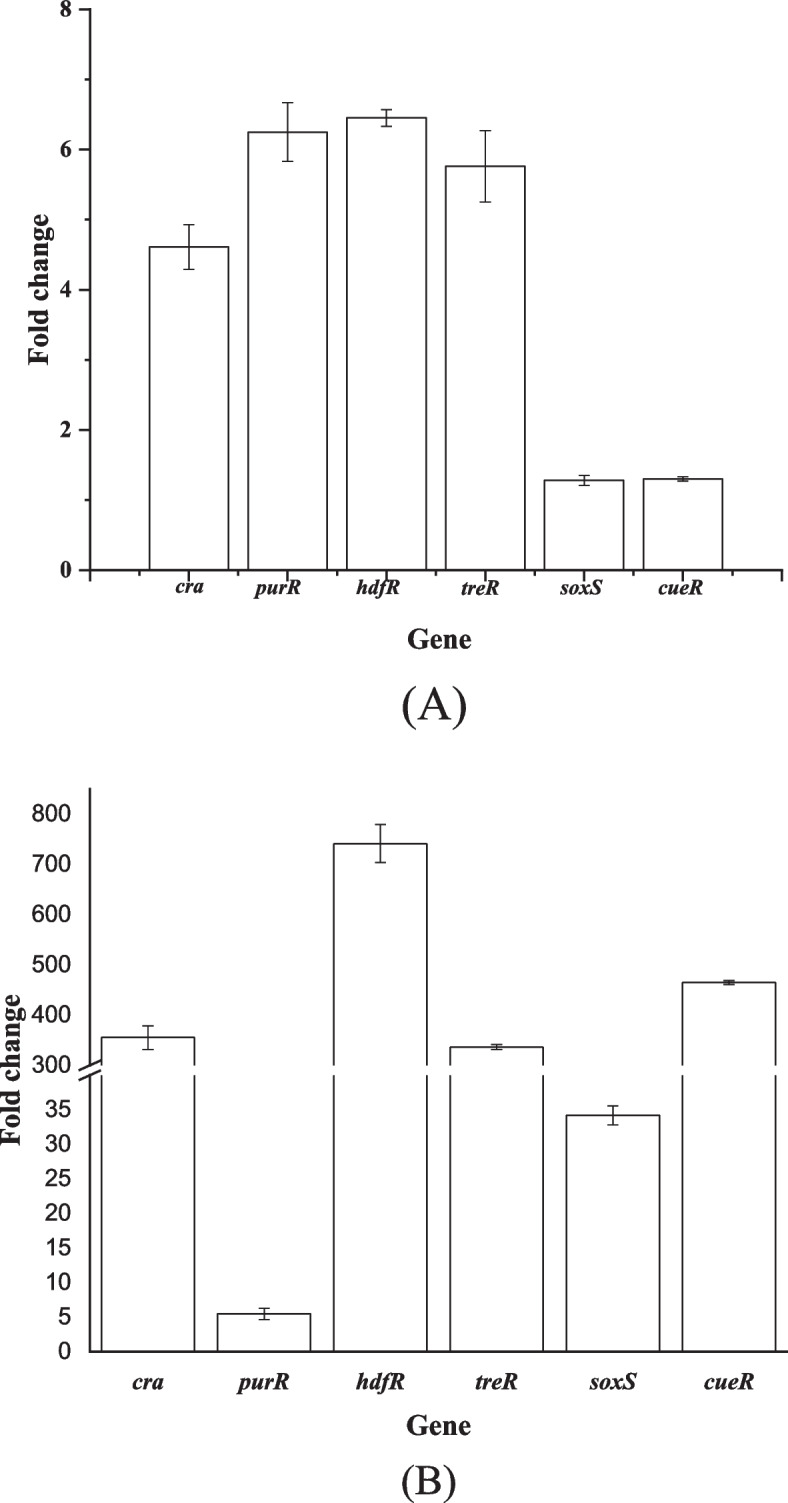
Relative transcriptional levels of the selected transcription factors in the CRISPRa activating (**A**) and promoter replacing strain (**B**) compared with those of the parent strain *E. coli* PHE02

Finally, we compared the changes in the transcriptional levels of the selected transcription factors after CRISPRa and replacing the native promoters. As shown in Fig. 3, the transcriptional levels of *cra*, *purR*, *hdfR*, *treR*, *soxS* and *cueR* were upregulated by 1.3- to 6.5-fold after CRISPRa activating. The transcriptional levels of *cra*, *purR*, *hdfR*, *treR*, *soxS* and *cueR* were upregulated by 5.5- to 741-fold after replacing the native promoters. These results demonstrate that the CRISPRa activating and replacing the native promoter of the selected transcription factors indeed increased the transcriptional levels of them. The transcriptional level of the same transcription factor in the promoter replacing strain was significantly higher than that in the CRISPRa activating strain. Table 2 shows that the CRISPRa activation of all 8 transcription factors increased the production of phenyllactic acid. However, the promoter replacement of only 3 transcription factors increased the production of phenyllactic acid (Table 3). This may be due to the different transcriptional levels in the promoter replacing strain and CRISPRa activating strain. Promoter engineering and 5’-untranslational region (UTR) engineering are a common strategy to control the transcription of genes. Optimizing the expression of the other 3 transcription factors (*cra, purR* and *hdfR*) may increase the production of phenyllactic acid by altering the promoter or UTR sequence.

To characterize the robustness of strains, we compared the production of phenyllactic acid in different scales of culture. As shown in Table 4 and Supplementary Table 4, the titer of phenyllactic acid in a 2 L shake flask culture (200 mL culture medium) was similar to that in a 250 mL shake flask culture (50 mL culture medium). This indicates that the promoter replacement of the 4 transcription factors (*cra, treR, soxS* and *cueR*) increased the strain robustness for scale-up culture.

**Table 4:** Comparing the production of phenyllactic acid in different scales of culture

Host strain	Plasmid	Phenyllactic acid (g/L)	
		250 mL	2 L
PHE02	pZBK-P_esaR_-Cn*ldhA*	4.95 ± 0.05	4.79 ± 0.02
PHE02(P_cra_::P37)		4.93 ± 0.13	5.31 ± 0.00
PHE02(P_treR_::P37)		5.39 ± 0.03	4.60 ± 0.01
PHE02(P_soxS_::P37)		5.47 ± 0.09	5.47 ± 0.02
PHE02(P_cueR_::P37)		5.43 ± 0.03	5.73 ± 0.21

### Screening transcription factors to increase the robustness to other aromatic chemicals

**Fig. 4:**
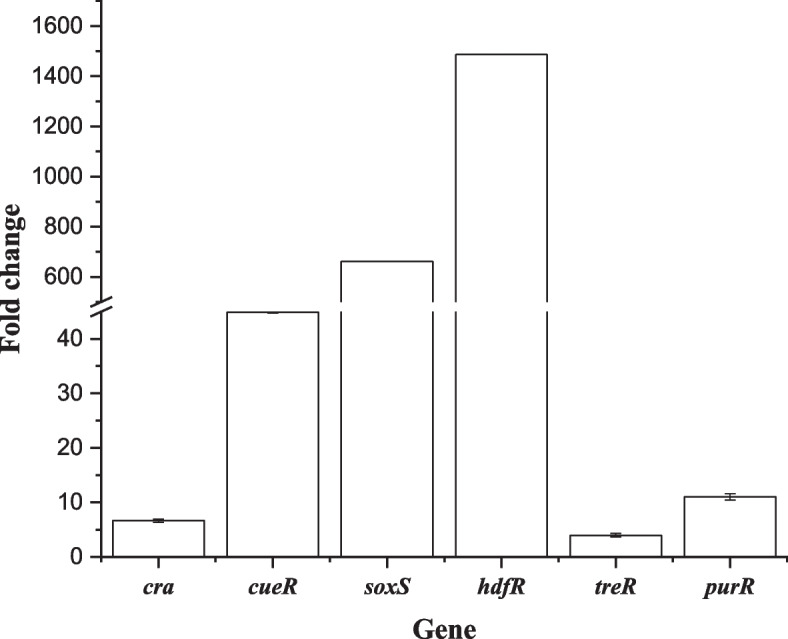
Relative transcriptional levels of the selected transcription factors in the promoter replacement strain compared with those of the parent strain *E. coli* TYR-14B1

Aromatic compounds are an important class of chemicals widely used in chemical, pharmaceutical and food industries. Many metabolically engineered microorganisms have been developed for the biotechnological production of aromatic chemicals from renewable sugar feedstocks (Shen et al. 2020). However, the high toxicity of aromatic chemicals limits their commercial production. Aromatic chemicals includes L-tryptophan, L-phenylalanine (for example, phenyllactic acid) and L-tyrosine derivatives. We engineered some *E. coli* for the production of L-tyrosine derivatives (Niu et al. 2020). Do the selected transcription factors influence the robustness to L-tyrosine derivatives? Of these L-tyrosine derivatives, caffeic acid and tyrosol (Fig. S4) show higher toxicity to *E. coli* (Fig. S2). Thus, we investigated the effect of the above transcription factors on the robustness to caffeic acid or tyrosol. We first replaced the native promoter of the selected transcription factors of the L-tyrosine-producing strain *E. coli* TYR-14B1 to obtain a responsive replacing strain. Then we determined the transcriptional levels of the selected transcription factors in the promoter replacement strain. As shown in Fig. 4, the replacement of the native promoter of the selected transcription factors in *E. coli* TYR-14B1 with the P37 promoter indeed upregulated their transcriptional levels by 4.0-fold to 1486-fold. These results demonstrate that replacing the native promoter of the selected transcription factors indeed increased their transcriptional levels.

The biosynthetic pathway of the L-tyrosine derivatives, such as caffeic acid, and tyrosol, was transferred into the promoter replacement strain, respectively. The robustness of these L-tyrosine derivatives-producing strains was tested. As shown in Table 5, the upregulation of *purR* and *treR* significantly improved the resistance to caffeic acid. Upregulation of other transcription factors did not show a significant effect on the resistance to caffeic acid. Upregulation of *cra* increased the production of caffeic acid. Upregulation of *cueR* did not show a significant effect on the production of caffeic acid. Upregulation of *purR*, *soxS, treR* and *hdfR* had a significant negative effect on the production of caffeic acid. After scaling up of culture from 250 mL to 2 L, the production of caffeic acid in the promoter replacing strain of *cra* did not show a significant difference (Supplementary Table 4). However, the production of caffeic acid in parent strain TYR14B1 (pZEA-RgTAL-PaHpaB-SeHpaC) significantly decreased. These results indicate that upregulation of *cra* increased the robustness to caffeic acid.

**Table 5:** Effects of the replacement of the promoter of the selected transcription factors on the resistance to caffeic acid and the production of caffeic acid

Host strain	Resistance^#^	Production of caffeic acid^##^		Yield (mg/g)
	OD_600_	OD_600_	Caffeic acid (mg/L)	
TYR-14B1	0.67 ± 0.00	8.20 ± 0.60	752.6 ± 17.1	47.7 ± 0.1
TYR-14B1 (P_cueR_::P37)	0.66 ± 0.00^n.s^	7.80 ± 0.30	765.1 ± 49.7^n.s^	40.6 ± 0.4
TYR-14B1 (P_cra:_:P37)	0.69 ± 0.01^n.s^	8.10 ± 0.50	875.3 ± 4.8*	55.1 ± 0.1
TYR-14B1 (P_purR_::P37)	0.76 ± 0.00**	10.90 ± 0.20	665.9 ± 4.4*	46.4 ± 0.1
TYR-14B1 (P_soxS_::P37)	0.65 ± 0.01^n.s^	4.20 ± 0.20	512.3 ± 42.8*	25.9 ± 0.1
TYR-14B1 (P_treR_::P37)	0.78 ± 0.01**	6.60 ± 0.10	428.7 ± 3.6*	23.9 ± 0.2
TYR-14B1 (P_hdfR_::P37)	0.65 ± 0.01^n.s^	3.60 ± 0.10	244.3 ± 2.9***	14.9 ± 0.1

Significance was performed by two-tailed *t*-test: not significant (n.s.), *p* ≤ 0.05 (*), *p* ≤ 0.01 (**), and *p* ≤ 0.001 (***).^#^Grown in LB + 10 g/L caffeic acid for 24 h at 37 °C, 200 rpm.^##^ *E. coli* was cultured for 72 h at 37 °C, 200 rpm

We investigated the effect of the upregulation of these transcription factors on the robustness to tyrosol. As shown in Table 6, the replacement of the native promoter of *cra, cueR, treR, soxS, hdfR* and *purR* improved the resistance to tyrosol. The replacement of the native promoter of all 6 transcription factors increased the production of tyrosol. After scaling up of culture from 250 mL to 2 L, the production of caffeic acid in the promoter replacing strain of *purR* and *hdfR* did not show a significant difference (Supplementary Table 4). However, the production of caffeic acid in parent strain TYR14B1 (pZEA(U)-SpyTag-aro10-linker-yahK) significantly decreased. These results indicate that the replacement of the native promoter of these transcription factors increased the robustness to tyrosol.

**Table 6:** Effects of the replacement of the promoter of the selected transcription factors on the resistance to tyrosol and the production of tyrosol

Host strain	Resistance^#^	Production of tyrosol^##^		Yield
	OD_600_	OD_600_	Tyrosol (g/L)	(mg/g)
TYR-14B1	0.20 ± 0.01	4.24 ± 0.15	0.70 ± 0.04	24.7 ± 1.3
TYR-14B1 (P_cra_::P37)	0.26 ± 0.05**	5.26 ± 0.03	1.93 ± 0.01***	54.5 ± 0.9
TYR-14B1 (P_cueR:_:P37)	0.21 ± 0.01^n.s^	5.23 ± 0.17	2.08 ± 0.02***	56.3 ± 0.3
TYR-14B1 (P_treR_::P37)	0.27 ± 0.01**	5.17 ± 0.15	1.40 ± 0.06***	38.8 ± 0.1
TYR-14B1 (P_soxS_::P37)	0.19 ± 0.01^n.s^	4.77 ± 0.03	2.22 ± 0.06***	55.9 ± 0.1
TYR-14B1 (P_hdfR_::P37)	0.25 ± 0.02**	5.90 ± 0.26	2.75 ± 0.08***	70.5 ± 0.1
TYR-14B1 (P_purR_::P37)	0.24 ± 0.02*	6.28 ± 0.08	2.44 ± 0.01***	61.0 ± 0.1

Significance was performed by two-tailed *t*-test: not significant (n.s.), *p* ≤ 0.05 (*), *p* ≤ 0.01 (**), and *p* ≤ 0.001 (***). ^#^ Grown in LB + 8 g/L tyrosol for 24 h at 37 °C, 200 rpm. ^##^ *E. coli* was cultured for 48 h at 30 °C, 200 rpm

In this study, we mined some transcription factors (*hdfR, yldP, purR, sosS, ygeH, cueR, cra,* and *treR*) that were related to the robustness of *E. coli* to aromatic chemicals using CRISPRa screening. Previous studies have reported transcription factors associated with robustness, including *σ*^*70*^ (Alper and Stephanopoulos 2007), *CRP* (Zhang et al. 2012; Chong et al. 2014), *DR1558* (Guo et al. 2017), *irrE* (Chen et al. 2011) and *Hass1* (Swinnen et al.^2017^). To our knowledge, this is the time that these transcription factors have been reported to be associated with strain robustness. Some transporters have been reported to be related to strain robustness. Overexpression of the phenolic acid efflux pump *aaeXAB* in *E. coli* improved the robustness to *p*-coumaric acid (Sariaslani 2007). Transcriptomic analysis of *E. coli* under caffeic acid and ferulic acid stress was used to mine robustness-associated transporters (Wang et al. 2023). Overexpression of the sugar ABC transporter permease *yciP* improved the caffeic acid titer. It was also reported that the transport protein Esbp 6 from *S. cerevisiae* was related to its robustness to aromatic compounds (*p*-coumaric acid and ferulic acid) (Pereira et al. 2020).

Although some transcription factors were identified using CRISPRa screening, many transcription factors reduced growth during CRISPRa screening (Fig. S3). Thus, more robustness-related transcription factors may be mined using CRISPR interference (CRISPRi) screening.

This study shows that only one transcription factor Cra is associated with strain robustness to caffeic acid. More robustness-related transcription factors may be mined by screening all transcription factors. In addition, to summarise the rule between transcription factors and the structure of aromatic compounds, robustness-associated transcription factors of more aromatic compounds should be mined.

## Conclusions

Transcription factors are key proteins controlling the expression of the responsive genes. Transcription factor engineering is becoming a feasible and efficient approach to improve strain robustness. In this study, we developed an approach to screen endogenous transcription factors to improve robustness using CRSPRa technology. We applied this approach to identify some transcription factors to increase the robustness of *E. coli* to aromatic chemicals. Activation of *hdfR, yldP, purR, sosS, ygeH, cueR, cra,* and *treR* increased the robustness to phenyllactic acid. These transcription factors may be used to increase robustness to phenyllactic acid. Promoter replacement further showed that upregulation of *treR, soxS* and *cueR* increased the robustness to phenyllactic acid. We also found that replacing the native promoter of some transcription factors increased the robustness to caffeic acid or tyrosol. Upregulation of *cra* increased the robustness to caffeic acid, indicating that this transcription factor was related to the increased robustness to caffeic acid. The *cra, cueR, treR, soxS, hdfR* and *purR* transcription factors were related to the increased robustness of tyrosol. Our study demonstrated that transcription factor engineering using CRISPRa is a powerful method to increase microbial robustness. We can also use CRISPRa screening technology to identify other endogenous genes, such as stress proteins and transporters, etc., to improve microbial resistance and robustness.

## Supplementary Information

**Additional file 1.** Supplementary data.

## Data Availability

Data sharing not applicable to this article as no datasets were generated or analyzed during the current study.
