# Supplementary material for: Increasing the robustness of Escherichia coli for aromatic chemicals production through transcription factor engineering
Source: Adv Biotechnol (Singap). 2024 Apr 2;2(2):15. doi: 10.1007/s44307-024-00023-x (PMC11740835; doi:10.1007/s44307-024-00023-x)
Supplement: Supplementary file 1 — Additional file 1. Supplementary data. [file 44307_2024_23_MOESM1_ESM.docx]

**Increasing the robustness of *Escherichia coli* to aromatic chemicals through transcription factor engineering**

Xiao-Ling Zhou^1^, Meng-Sang Zhang^1^, Xing-Run Zheng^1^, Zhi-Qian Zhang^2,3^ and Jian-Zhong Liu^1,2^*

1. State key laboratory of biocontrol, School of Life Sciences, Sun Yat-Sen University, Guangzhou 510275, China

2. Joint research center of engineering biotechnology of Sun Yat-sen University and Tidetron, Guangzhou 510275, China

3. Tidetron Bioworks Technology (Guangzhou) Co., Ltd. Guangzhou 510000, China

Email:

Xiao-Ling Zhou: zhouxling3@mail2.sysu.edu.cn

Meng-Sang Zhang: zhangms3@mail2.sysu.edu.cn

Xing-Run Zheng: zhengxr35@mail2.sysu.edu.cn

Zhi-Qian Zhang: worthy@tidetronbio.com

Jian-Zhong Liu: lssljz@mail.sysu.edu.cn

* Corresponding author: School of Life Science, Sun Yat-Sen University, Guangzhou 510275, P.R. China.

**Table S1 Primers used in this study**

| Name | 5’-3’ Sequence | Purpose |
| --- | --- | --- |
| For CRISPRa |  |  |
| KpnⅠ-pTargetA-R | GGGGTACCttctctatcactgatagggagtggtaaaataactctatcaa | for pTargetA |
| *Kpn*Ⅰ-pTargetA-*araC* | GGGGTACCtcttatggataaaaatgctagttttagagctagaaatag | for *araC* scRNA |
| *Kpn*Ⅰ-pTargetA-*rhaR/rhaS* | GGGGTACCaagatacagcgtgaattttcgttttagagctagaaatag | for *rhaR/rhaS* scRNA |
| *Kpn*Ⅰ-pTargetA-*chbR* | GGGGTACCgtgaagagggtcataaccacgttttagagctagaaatag | for *chbR* scRNA |
| *Kpn*Ⅰ-pTargetA-*adiY* | GGGGTACCaggcaaaaaaaagcgcgggggttttagagctagaaatag | for *adiY* scRNA |
| *Kpn*Ⅰ-pTargetA-*appY* | GGGGTACCtatatatagcaagaggtttcgttttagagctagaaatag | for *appY* scRNA |
| *Kpn*Ⅰ-pTargetA-*envY* | GGGGTACCcacaatgcgctatctgttttgttttagagctagaaatag | for *envY* scRNA |
| *Kpn*Ⅰ-pTargetA-*soxS* | GGGGTACCattaaagcgctgctaaccccgttttagagctagaaatag | for *soxS* scRNA |
| *Kpn*Ⅰ-pTargetA-*marA* | GGGGTACCgttttgttcaatgcgatgcagttttagagctagaaatag | for *marA* scRNA |
| *Kpn*Ⅰ-pTargetA-*rob* | GGGGTACCcacgagcaattagttcgtcagttttagagctagaaatag | for *rob* scRNA |
| *Kpn*Ⅰ-pTargetA-*feaR* | GGGGTACCgtcaggcatagagacataaggttttagagctagaaatag | for *feaR* scRNA |
| *Kpn*Ⅰ-pTargetA-*gadW* | GGGGTACCtgttatgcaaaactactttggttttagagctagaaatag | for *gadW* scRNA |
| *Kpn*Ⅰ-pTargetA-*gadX* | GGGGTACCtgataatgtgtgatgttctagttttagagctagaaatag | for *gadX* scRNA |
| *Kpn*Ⅰ-pTargetA-*melR* | GGGGTACCggagagtcccctcgcccctcgttttagagctagaaatag | for *melR* scRNA |
| *Kpn*Ⅰ-pTargetA-*ada* | GGGGTACCacaaggaagcttttaaactggttttagagctagaaatag | for *ada* scRNA |
| *Kpn*Ⅰ-pTargetA- *eutR* | GGGGTACCgagctgttcgagcgcatttcgttttagagctagaaatag | for *eutR* scRNA |
| *Kpn*Ⅰ-pTargetA-*rclR* | GGGGTACCtcaaatgaacgcgtttcgacgttttagagctagaaatag | for *rclR* scRNA |
| *Kpn*Ⅰ-pTargetA-*gcvA* | GGGGTACCgccggaacgaaaagttttatgttttagagctagaaatag | for *gcvA* scRNA |
| *Kpn*Ⅰ-pTargetA-*ilvY* | GGGGTACCatgttgtgcttcttattgttgttttagagctagaaatag | for *ilvY* scRNA |
| *Kpn*Ⅰ-pTargetA-*metR* | GGGGTACCtccttcgtcttttaaatttagttttagagctagaaatag | for *metR* scRNA |
| *Kpn*Ⅰ-pTargetA-*nhaR* | GGGGTACCataattaatttgttgtcagtgttttagagctagaaatag | for *nhaR* scRNA |
| *Kpn*Ⅰ-pTargetA-*oxyR* | GGGGTACCgtgacttcaagggttaaaaggttttagagctagaaatag | for *oxyR* scRNA |
| *Kpn*Ⅰ-pTargetA-*argP* | GGGGTACCttaagctgcgcttttaaccagttttagagctagaaatag | for *argP* scRNA |
| *Kpn*Ⅰ-pTargetA-*leuO* | GGGGTACCatactcaatcatctacaaaagttttagagctagaaatag | for *leuO* scRNA |
| *Kpn*Ⅰ-pTargetA-*hcaR* | GGGGTACCaatgttcaaatctgagggtggttttagagctagaaatag | for *hcaR* scRNA |
| *Kpn*Ⅰ-pTargetA-*tdcA* | GGGGTACCtaattgtaataacgatactcgttttagagctagaaatag | for *tdcA* scRNA |
| *Kpn*Ⅰ-pTargetA-*allS* | GGGGTACCgtcagcttggtttcaataacgttttagagctagaaatag | for *allS* scRNA |
| *Kpn*Ⅰ-pTargetA-*cynR* | GGGGTACCctcattccagagacagacaggttttagagctagaaatag | for *cynR* scRNA |
| *Kpn*Ⅰ-pTargetA-*cysB* | GGGGTACCaaataatatgcaataaaaaagttttagagctagaaatag | for *cysB* scRNA |
| *Kpn*Ⅰ-pTargetA-*cbl* | GGGGTACCtggtgagctaagtgttatatgttttagagctagaaatag | for *cbl* scRNA |
| *Kpn*Ⅰ-pTargetA-*dsdC* | GGGGTACCatatcatcaggttaatcacagttttagagctagaaatag | for *dsdC* scRNA |
| *Kpn*Ⅰ-pTargetA-*abgR* | GGGGTACCaaaactatcgctacgtgaacgttttagagctagaaatag | for *abgR* scRNA |
| *Kpn*Ⅰ-pTargetA-*dmlR* | GGGGTACCctttgccaatcccgtctcccgttttagagctagaaatag | for *dmlR* scRNA |
| *Kpn*Ⅰ-pTargetA-*nac* | GGGGTACCggaacgcgacagctgattaagttttagagctagaaatag | for *nac* scRNA |
| *Kpn*Ⅰ-pTargetA-*hypT* | GGGGTACCggcaaactgatggtcaaagagttttagagctagaaatag | for *hypT* scRNA |
| *Kpn*Ⅰ-pTargetA-*aaeR* | GGGGTACCacagcaatgttgctctcgccgttttagagctagaaatag | for *aaeR* scRNA |
| *Kpn*Ⅰ-pTargetA-*ttdR* | GGGGTACCcttattactttcgctcatcagttttagagctagaaatag | for *ttdR* scRNA |
| *Kpn*Ⅰ-pTargetA-*hdfR* | GGGGTACCccatgacgagagaatccacggttttagagctagaaatag | for *hdfR* scRNA |
| *Kpn*Ⅰ-pTargetA-*pgrR* | GGGGTACCaacgaaccgacaactccatggttttagagctagaaatag | for *pgrR* scRNA |
| *Kpn*Ⅰ-pTargetA-*deoR* | GGGGTACCtctatcaacgtctgcaatcggttttagagctagaaatag | for *deoR* scRNA |
| *Kpn*Ⅰ-pTargetA-*agaR* | GGGGTACCaaaaataaaacgaaagataagttttagagctagaaatag | for *agaR* scRNA |
| *Kpn*Ⅰ-pTargetA-*fucR* | GGGGTACCaatttcgattattaaagtgagttttagagctagaaatag | for *fucR* scRNA |
| *Kpn*Ⅰ-pTargetA-*srlR* | GGGGTACCgcgatggcgaccacgacccggttttagagctagaaatag | for *srlR* scRNA |
| *Kpn*Ⅰ-pTargetA-*ulaR* | GGGGTACCtcgaagttgtctttatccaggttttagagctagaaatag | for *ulaR* scRNA |
| *Kpn*Ⅰ-pTargetA-*galR* | GGGGTACCttgtcttacttatttgcgttgttttagagctagaaatag | for *galR* scRNA |
| *Kpn*Ⅰ-pTargetA-*xylR* | GGGGTACCtttaacgatatactgccagagttttagagctagaaatag | for *xylR* scRNA |
| *Kpn*Ⅰ-pTargetA-*rbsR* | GGGGTACCcaacctcgaaacgttttacagttttagagctagaaatag | for *rbsR* scRNA |
| *Kpn*Ⅰ-pTargetA-*galS* | GGGGTACCgatcacattcgttaacaaaagttttagagctagaaatag | for *galS* scRNA |
| *Kpn*Ⅰ-pTargetA-*cytR* | GGGGTACCgatccgcctcgcatcgtgaggttttagagctagaaatag | for *cytR* scRNA |
| *Kpn*Ⅰ-pTargetA-*cra* | GGGGTACCggtgtggtcggactttcgcggttttagagctagaaatag | for *cra* scRNA |
| *Kpn*Ⅰ-pTargetA-*treR* | GGGGTACCaataatattcgtagcatctagttttagagctagaaatag | for *treR* scRNA |
| *Kpn*Ⅰ-pTargetA-*ascG* | GGGGTACCattttttatatttgtgaaacgttttagagctagaaatag | for *ascG* scRNA |
| *Kpn*Ⅰ-pTargetA-*purR* | GGGGTACCgcgtaatatcactttttgtggttttagagctagaaatag | for *purR* scRNA |
| *Kpn*Ⅰ-pTargetA-*gntR* | GGGGTACCgcacgcgaccggagcgagcggttttagagctagaaatag | for *gntR* scRNA |
| *Kpn*Ⅰ-pTargetA-*idnR* | GGGGTACCgaaacccttatttctattatgttttagagctagaaatag | for *idnR* scRNA |
| *Kpn*Ⅰ-pTargetA-*ebgR* | GGGGTACCacgctgtaacggtaacacaggttttagagctagaaatag | for *ebgR* scRNA |
| *Kpn*Ⅰ-pTargetA-*malI* | GGGGTACCactcctcgtagagaacaatcgttttagagctagaaatag | for *malI* scRNA |
| *Kpn*Ⅰ-pTargetA-*yidP* | GGGGTACCagctagttagctatttaacggttttagagctagaaatag | for *yidP* scRNA |
| *Kpn*Ⅰ-pTargetA-*phnF* | GGGGTACCgtgcgggagggcgggcctccgttttagagctagaaatag | for *phnF* scRNA |
| *Kpn*Ⅰ-pTargetA-*frlR* | GGGGTACCacgccaacgttatacctaccgttttagagctagaaatag | for *frlR* scRNA |
| *Kpn*Ⅰ-pTargetA-*mngR* | GGGGTACCtattgttttatcgggcacacgttttagagctagaaatag | for *mngR* scRNA |
| *Kpn*Ⅰ-pTargetA-*fadR* | GGGGTACCtagtcaaggggaaagagatggttttagagctagaaatag | for *fadR* scRNA |
| *Kpn*Ⅰ-pTargetA-*pdhR* | GGGGTACCcacaaatgtagactttacgtgttttagagctagaaatag | for *pdhR* scRNA |
| *Kpn*Ⅰ-pTargetA-*glcC* | GGGGTACCtcgtctatctctttagctacgttttagagctagaaatag | for *glcC* scRNA |
| *Kpn*Ⅰ-pTargetA-*uxuR* | GGGGTACCtgattgaccagtaagtctgtgttttagagctagaaatag | for *uxuR* scRNA |
| *Kpn*Ⅰ-pTargetA-*exuR* | GGGGTACCgttcgacctgttaggtgcgcgttttagagctagaaatag | for *exuR* scRNA |
| *Kpn*Ⅰ-pTargetA-*lldR* | GGGGTACCttcttcgcttatctgacctcgttttagagctagaaatag | for *lldR* scRNA |
| *Kpn*Ⅰ-pTargetA-*dgoR* | GGGGTACCtttgtactacattacacgcagttttagagctagaaatag | for *dgoR* scRNA |
| *Kpn*Ⅰ-pTargetA-*nanR* | GGGGTACCcgtgtttttaattgtaggtggttttagagctagaaatag | for *nanR* scRNA |
| *Kpn*Ⅰ-pTargetA-*mcbR* | GGGGTACCctgctgaagggtaaaaacttgttttagagctagaaatag | for *mcbR* scRNA |
| *Kpn*Ⅰ-pTargetA-*csiR* | GGGGTACCaatccctatcgctaataagcgttttagagctagaaatag | for *csiR* scRNA |
| *Kpn*Ⅰ-pTargetA-*ydfH* | GGGGTACCcattcagtccggcatagcttgttttagagctagaaatag | for *ydfH* scRNA |
| *Kpn*Ⅰ-pTargetA-*lgoR* | GGGGTACCagcagccgaaagaattagtcgttttagagctagaaatag | for *lgoR* scRNA |
| *Kpn*Ⅰ-pTargetA-*betI* | GGGGTACCttaaatagtaacaataacaggttttagagctagaaatag | for *betI* scRNA |
| *Kpn*Ⅰ-pTargetA-*acrR* | GGGGTACCttacatatgaacaaaaacaggttttagagctagaaatag | for *acrR* scRNA |
| *Kpn*Ⅰ-pTargetA-*slmA* | GGGGTACCcgttcgcgcagatcagatcagttttagagctagaaatag | for *slmA* scRNA |
| *Kpn*Ⅰ-pTargetA-*rutR* | GGGGTACCtttgcaaacaagccaatacggttttagagctagaaatag | for *rutR* scRNA |
| *Kpn*Ⅰ-pTargetA-*nemR* | GGGGTACCaatggtaataatcttatctggttttagagctagaaatag | for *nemR* scRNA |
| *Kpn*Ⅰ-pTargetA-*uidR* | GGGGTACCatttcagagtgaaaagtatagttttagagctagaaatag | for *uidR* scRNA |
| *Kpn*Ⅰ-pTargetA-*envR* | GGGGTACCctactgttttattttgacgcgttttagagctagaaatag | for *envR* scRNA |
| *Kpn*Ⅰ-pTargetA-*bdcR* | GGGGTACCaagacagttctcatcctcgggttttagagctagaaatag | for *bdcR* scRNA |
| *Kpn*Ⅰ-pTargetA-*comR* | GGGGTACCtagaaagcaattatttttatgttttagagctagaaatag | for *comR* scRNA |
| *Kpn*Ⅰ-pTargetA-*fabR* | GGGGTACCacatatttactgcaattcttgttttagagctagaaatag | for *fabR* scRNA |
| *Kpn*Ⅰ-pTargetA-*cecR* | GGGGTACCgcataaattatccctgtgacgttttagagctagaaatag | for *cecR* scRNA |
| *Kpn*Ⅰ-pTargetA-*rcdA* | GGGGTACCtgcattacgtgaagaatttagttttagagctagaaatag | for *rcdA* scRNA |
| *Kpn*Ⅰ-pTargetA-*asnC* | GGGGTACCgttgcttaatcataagcaacgttttagagctagaaatag | for *asnC* scRNA |
| *Kpn*Ⅰ-pTargetA-*lrp* | GGGGTACCcatgccagatattcattaacgttttagagctagaaatag | for *lrp* scRNA |
| *Kpn*Ⅰ-pTargetA-*decR* | GGGGTACCaaattcaacgcaaaaaaaacgttttagagctagaaatag | for *decR* scRNA |
| *Kpn*Ⅰ-pTargetA-*malT* | GGGGTACCgtgagttgttaataaagattgttttagagctagaaatag | for *malT* scRNA |
| *Kpn*Ⅰ-pTargetA-*csgD* | GGGGTACCatattaaaatgttaaccttagttttagagctagaaatag | for *csgD* scRNA |
| *Kpn*Ⅰ-pTargetA-*rcsA* | GGGGTACCtaaaacaaatatttcgctgcgttttagagctagaaatag | for *rcsA* scRNA |
| *Kpn*Ⅰ-pTargetA-*sdiA* | GGGGTACCatttgttaagcagttttatcgttttagagctagaaatag | for *sdiA* scRNA |
| *Kpn*Ⅰ-pTargetA-*ecpR* | GGGGTACCttcacatattgacactcatcgttttagagctagaaatag | for *ecpR* scRNA |
| *Kpn*Ⅰ-pTargetA-*dctR* | GGGGTACCaaaggatataaacatcagacgttttagagctagaaatag | for *dctR* scRNA |
| *Kpn*Ⅰ-pTargetA-*bglJ* | GGGGTACCgattttgcattctgcgggaagttttagagctagaaatag | for *bglJ* scRNA |
| *Kpn*Ⅰ-pTargetA-*gadE* | GGGGTACCacgaagggtaaagttcttatgttttagagctagaaatag | for *gadE* scRNA |
| *Kpn*Ⅰ-pTargetA-*marR* | GGGGTACCgttttgttcaatgcgatgcagttttagagctagaaatag | for *marR* scRNA |
| *Kpn*Ⅰ-pTargetA-*slyA* | GGGGTACCtcagacatatttttcagaaagttttagagctagaaatag | for *slyA* scRNA |
| *Kpn*Ⅰ-pTargetA-*mprA* | GGGGTACCtataatcgccatcactttccgttttagagctagaaatag | for *mprA* scRNA |
| *Kpn*Ⅰ-pTargetA-*cueR* | GGGGTACCgaccgccaccacaataatacgttttagagctagaaatag | for *cueR* scRNA |
| *Kpn*Ⅰ-pTargetA-*zntR* | GGGGTACCtagccgtcgattgtcattacgttttagagctagaaatag | for *zntR* scRNA |
| *Kpn*Ⅰ-pTargetA-*soxR* | GGGGTACCgtcccatcagaaaattattcgttttagagctagaaatag | for *soxR* scRNA |
| *Kpn*Ⅰ-pTargetA-*mlrA* | GGGGTACCagggtgattttatttttgccgttttagagctagaaatag | for *mlrA* scRNA |
| *Kpn*Ⅰ-pTargetA-*bluR* | GGGGTACCgctgaaaattattttttctcgttttagagctagaaatag | for *bluR* scRNA |
| *Kpn*Ⅰ-pTargetA-*fur* | GGGGTACCggcacagcttacatttacaagttttagagctagaaatag | for *fur* scRNA |
| *Kpn*Ⅰ-pTargetA-*zur* | GGGGTACCtaactcgctatttatttaacgttttagagctagaaatag | for *zur* scRNA |
| *Kpn*Ⅰ-pTargetA-*iclR* | GGGGTACCtgaacaaatctcatgttgcggttttagagctagaaatag | for *iclR* scRNA |
| *Kpn*Ⅰ-pTargetA-*mhpR* | GGGGTACCcgagtcgtgaggtactgaaagttttagagctagaaatag | for *mhpR* scRNA |
| *Kpn*Ⅰ-pTargetA-*allR* | GGGGTACCtgttgaaagtattcctgaacgttttagagctagaaatag | for *allR* scRNA |
| *Kpn*Ⅰ-pTargetA-*kdgR* | GGGGTACCgtcgcagaattgtaactaacgttttagagctagaaatag | for *kdgR* scRNA |
| *Kpn*Ⅰ-pTargetA-*yiaJ* | GGGGTACCtgtttttttgatcgctttttgttttagagctagaaatag | for *yiaJ* scRNA |
| *Kpn*Ⅰ-pTargetA-*crp* | GGGGTACCtcgccttccgttgcgcgattgttttagagctagaaatag | for *crp* scRNA |
| *Kpn*Ⅰ-pTargetA-*fnr* | GGGGTACCcatatatttacatctaatatgttttagagctagaaatag | for *fnr* scRNA |
| *Kpn*Ⅰ-pTargetA-*yeiL* | GGGGTACCtgtagaaaaaataacgcaacgttttagagctagaaatag | for *yeiL* scRNA |
| *Kpn*Ⅰ-pTargetA-*iscR* | GGGGTACCcgataaaaaaatggcactgagttttagagctagaaatag | for *iscR* scRNA |
| *Kpn*Ⅰ-pTargetA-*nsrR* | GGGGTACCgaacgggtcgcgcagaatcagttttagagctagaaatag | for *nsrR* scRNA |
| *Kpn*Ⅰ-pTargetA-*arsR* | GGGGTACCtaagtgcagaggaaggtaatgttttagagctagaaatag | for *arsR* scRNA |
| *Kpn*Ⅰ-pTargetA-*sutR* | GGGGTACCcccgccagtagcgtgggtgggttttagagctagaaatag | for *sutR* scRNA |
| *Kpn*Ⅰ-pTargetA-*mntR* | GGGGTACCatcgacgggattagcaagtcgttttagagctagaaatag | for *mntR* scRNA |
| *Kpn*Ⅰ-pTargetA-*alsR* | GGGGTACCgtaactctgtaaaacagatcgttttagagctagaaatag | for *alsR* scRNA |
| *Kpn*Ⅰ-pTargetA-*murR* | GGGGTACCgcgctcaacggcaagcggtagttttagagctagaaatag | for *murR* scRNA |
| *Kpn*Ⅰ-pTargetA-*yebK* | GGGGTACCagtgtcagatttttacccaagttttagagctagaaatag | for *yebK* scRNA |
| *Kpn*Ⅰ-pTargetA-*birA* | GGGGTACCacgcaaaaaggccatccgtcgttttagagctagaaatag | for *birA* scRNA |
| *Kpn*Ⅰ-pTargetA-*fis* | GGGGTACCaattgcatttaaaatgagcggttttagagctagaaatag | for *fis* scRNA |
| *Kpn*Ⅰ-pTargetA-*trpR* | GGGGTACCttactaccgctatttcatgggttttagagctagaaatag | for *trpR* scRNA |
| *Kpn*Ⅰ-pTargetA-*prpR* | GGGGTACCttaactgaaacgcatatttggttttagagctagaaatag | for *prpR* scRNA |
| *Kpn*Ⅰ-pTargetA-*sfsB* | GGGGTACCgatgcacatccctatttttcgttttagagctagaaatag | for *sfsB* scRNA |
| *Kpn*Ⅰ-pTargetA-*sgrR* | GGGGTACCccagcacaacttcgctgtcggttttagagctagaaatag | for *sgrR* scRNA |
| *Kpn*Ⅰ-pTargetA-*nadR* | GGGGTACCatcccttccggcgcttttttgttttagagctagaaatag | for *nadR* scRNA |
| *Kpn*Ⅰ-pTargetA-*mqsA* | GGGGTACCgatagtatgatcagagtagggttttagagctagaaatag | for *mqsA* scRNA |
| *Kpn*Ⅰ-pTargetA-*puuR* | GGGGTACCagtttgcatacagattcgaagttttagagctagaaatag | for *puuR* scRNA |
| *Kpn*Ⅰ-pTargetA-*hipB* | GGGGTACCcgctgcgatatccgcgatcggttttagagctagaaatag | for *hipB* scRNA |
| *Kpn*Ⅰ-pTargetA-*dicA* | GGGGTACCttagttatgactaacaataagttttagagctagaaatag | for *dicA* scRNA |
| *Kpn*Ⅰ-pTargetA-*higA* | GGGGTACCaaacaagagaatgtagacgagttttagagctagaaatag | for *higA* scRNA |
| *Kpn*Ⅰ-pTargetA-*hyfR* | GGGGTACCtctgaacggtacaaaaatgagttttagagctagaaatag | for *hyfR* scRNA |
| *Kpn*Ⅰ-pTargetA-*norR* | GGGGTACCaaaacaatcagataaaaaacgttttagagctagaaatag | for *norR* scRNA |
| *Kpn*Ⅰ-pTargetA-*fhlA* | GGGGTACCgtcgaaatgacacgtcgacagttttagagctagaaatag | for *fhlA* scRNA |
| *Kpn*Ⅰ-pTargetA-*cdaR* | GGGGTACCggtgaagttcacagattgtagttttagagctagaaatag | for *cdaR* scRNA |
| *Kpn*Ⅰ-pTargetA-*modE* | GGGGTACCaatagtctcatgactatatcgttttagagctagaaatag | for *modE* scRNA |
| *Kpn*Ⅰ-pTargetA-*yiaG* | GGGGTACCtataagtcacgctaaatgacgttttagagctagaaatag | for *yiaG* scRNA |
| *Kpn*Ⅰ-pTargetA-*nikR* | GGGGTACCcgggtaatggttatggacaagttttagagctagaaatag | for *nikR* scRNA |
| *Kpn*Ⅰ-pTargetA-*metJ* | GGGGTACCatataattttaacggctattgttttagagctagaaatag | for *metJ* scRNA |
| *Kpn*Ⅰ-pTargetA-*putA* | GGGGTACCataaaagaaatcgatatgacgttttagagctagaaatag | for *putA* scRNA |
| *Kpn*Ⅰ-pTargetA-*relB* | GGGGTACCatcactgtccgctttgtgccgttttagagctagaaatag | for *relB* scRNA |
| *Kpn*Ⅰ-pTargetA-*bglG* | GGGGTACCtattaactttgtgtaattttgttttagagctagaaatag | for *bglG* scRNA |
| *Kpn*Ⅰ-pTargetA-*frvR* | GGGGTACCttggcgtgcagataacgagtgttttagagctagaaatag | for *frvR* scRNA |
| *Kpn*Ⅰ-pTargetA-*frmR* | GGGGTACCaatgtggtcggaaaacaaaggttttagagctagaaatag | for *frmR* scRNA |
| *Kpn*Ⅰ-pTargetA-*rcnR* | GGGGTACCatccccagcgccatcttactgttttagagctagaaatag | for *rcnR* scRNA |
| *Kpn*Ⅰ-pTargetA-*bolA* | GGGGTACCagtaaacttcatacgcttgagttttagagctagaaatag | for *bolA* scRNA |
| *Kpn*Ⅰ-pTargetA-*rfaH* | GGGGTACCcaaagatgaacgccgtataagttttagagctagaaatag | for *rfaH* scRNA |
| *Kpn*Ⅰ-pTargetA-*argR* | GGGGTACCttatcaaattgatgttgtttgttttagagctagaaatag | for *argR* scRNA |
| *Kpn*Ⅰ-pTargetA-*nagC* | GGGGTACCgaaaggcgacaaactgtgtcgttttagagctagaaatag | for *nagC* scRNA |
| *Kpn*Ⅰ-pTargetA-*mlc* | GGGGTACCgtgattaacagcacatttttgttttagagctagaaatag | for *mlc* scRNA |
| *Kpn*Ⅰ-pTargetA-*mtlR* | GGGGTACCcataaatatagggggtagaggttttagagctagaaatag | for *mtlR* scRNA |
| *Kpn*Ⅰ-pTargetA-*tyrR* | GGGGTACCagacggaaaaacgggcacttgttttagagctagaaatag | for *tyrR* scRNA |
| *Kpn*Ⅰ-pTargetA-*cadC* | GGGGTACCgtaatcaagaagtttatggcgttttagagctagaaatag | for *cadC* scRNA |
| *Kpn*Ⅰ-pTargetA-*ygeH* | GGGGTACCaatcatttggttaaatttgcgttttagagctagaaatag | for *ygeH* scRNA |
| *Kpn*Ⅰ-pTargetA-*gcvR* | GGGGTACCtctcaatggtacgtttggtagttttagagctagaaatag | for *gcvR* scRNA |
| *Kpn*Ⅰ-pTargetA-*paaX* | GGGGTACCtttgatctgcgcaatattgtgttttagagctagaaatag | for *paaX* scRNA |
| *Kpn*Ⅰ-pTargetA-*pspF* | GGGGTACCaaatcaaaaagataaaaaatgttttagagctagaaatag | for *pspF* scRNA |
| *Kpn*Ⅰ-pTargetA-*pspC* | GGGGTACCagaatcaacagcaacatgccgttttagagctagaaatag | for *pspC* scRNA |
| *Kpn*Ⅰ-pTargetA-*lsrR* | GGGGTACCatgctcgtagagtcaaactggttttagagctagaaatag | for *lsrR* scRNA |
| *Kpn*Ⅰ-pTargetA-*dhaR* | GGGGTACCttcccttgcctgatgcacaagttttagagctagaaatag | for *dhaR* scRNA |
| *Kpn*Ⅰ-pTargetA-*rnk* | GGGGTACCatacataatgcgctgcattggttttagagctagaaatag | for *rnk* scRNA |
| *Kpn*Ⅰ-pTargetA-*alpA* | GGGGTACCtctaatacattgatttatgggttttagagctagaaatag | for *alpA* scRNA |
| *Kpn*Ⅰ-pTargetA-*yqgE* | GGGGTACCctgctgtaaacgtgcttcgagttttagagctagaaatag | for *yqgE* scRNA |
| *Kpn*Ⅰ-pTargetA-*caiF* | GGGGTACCcccattaagtaaatcttttggttttagagctagaaatag | for *caiF* scRNA |
| *Kpn*Ⅰ-pTargetA-*dksA* | GGGGTACCtaactggttaatttacattcgttttagagctagaaatag | for *dksA* scRNA |
| *Kpn*Ⅰ-pTargetA-*nrdR* | GGGGTACCtgcttttaccctctaccattgttttagagctagaaatag | for *nrdR* scRNA |
| *Kpn*Ⅰ-pTargetA-*rtcR* | GGGGTACCaagctgcttatttaattttcgttttagagctagaaatag | for *rtcR* scRNA |
| *Kpn*Ⅰ-pTargetA-*dicC* | GGGGTACCgaaacaaaaaatttaactatgttttagagctagaaatag | for *dicC* scRNA |
| *Kpn*Ⅰ-pTargetA-*ygiV* | GGGGTACCtgaatctcaaccgtatctttgttttagagctagaaatag | for *ygiV* scRNA |
| *Kpn*Ⅰ-pTargetA-*rpoA* | GGGGTACCcttgcaaagttgggttgagcgttttagagctagaaatag | for *rpoA* scRNA |
| *Kpn*Ⅰ-pTargetA-*rpoB/rpoC* | GGGGTACCtgagaaatcaggctgatggcgttttagagctagaaatag | for *rpoB/rpoC* scRNA |
| *Kpn*Ⅰ-pTargetA-*rpoZ* | GGGGTACCgcggctcatgcgcagacgttgttttagagctagaaatag | for *rpoZ* scRNA |
| *Kpn*Ⅰ-pTargetA-*rpoD* | GGGGTACCatagtgctctaaaagttgccgttttagagctagaaatag | for *rpoD* scRNA |
| *Kpn*Ⅰ-pTargetA-*rpoS* | GGGGTACCaaaatgcaagcgtgttgaacgttttagagctagaaatag | for *rpoS* scRNA |
| *Kpn*Ⅰ-pTargetA-*rpoE* | GGGGTACCctgcctgaagagtaacccaagttttagagctagaaatag | for *rpoE* scRNA |
| *Kpn*Ⅰ-pTargetA-*fecI* | GGGGTACCttttctaagtgttataaggtgttttagagctagaaatag | for *fecI* scRNA |
| *Kpn*Ⅰ-pTargetA-*rpoH* | GGGGTACCtgaacttgtggataaaatcagttttagagctagaaatag | for *rpoH* scRNA |
| *Kpn*Ⅰ-pTargetA-*rpoN* | GGGGTACCgcgcttatatcgtcagtcaggttttagagctagaaatag | for *rpoN* scRNA |
| *Kpn*Ⅰ-pTargetA-*fliA* | GGGGTACCaggggttatttgggggttacgttttagagctagaaatag | for *fliA* scRNA |
| *Kpn*Ⅰ-pTargetA-*rapA* | GGGGTACCtcaaaatagcccaagttgccgttttagagctagaaatag | for *rapA* scRNA |
| *Kpn*Ⅰ-pTargetA-*nusA* | GGGGTACCcggtacaaaaccgtcgtaaagttttagagctagaaatag | for *nusA* scRNA |
| *Kpn*Ⅰ-pTargetA-*nusB* | GGGGTACCgcgtcgctgcctaatagtttgttttagagctagaaatag | for *nusB* scRNA |
| *Kpn*Ⅰ-pTargetA-*nusG* | GGGGTACCtgctacaacgcccgcgccaagttttagagctagaaatag | for *nusG* scRNA |
| *Kpn*Ⅰ-pTargetA-*sspA/sspB* | GGGGTACCtagttgacttagtcccttatgttttagagctagaaatag | for *sspA/sspB* scRNA |
| *Kpn*Ⅰ-pTargetA-*rseA/rseB/rseC* | GGGGTACCttcaatggcatccacatcacgttttagagctagaaatag | for *rseA/rseB/rseC* scRNA |
| *Kpn*Ⅰ-pTargetA-*dksA* | GGGGTACCggctcttaaaaaatcactgcgttttagagctagaaatag | for *dksA* scRNA |
| *Kpn*Ⅰ-pTargetA-*greA* | GGGGTACCtagtatgacggctcgattccgttttagagctagaaatag | for *greA* scRNA |
| *Kpn*Ⅰ-pTargetA-*greB* | GGGGTACCaaattcataccagatttagcgttttagagctagaaatag | for *greB* scRNA |
| *Kpn*Ⅰ-pTargetA-*rho* | GGGGTACCcttacgccaggttagcgtcggttttagagctagaaatag | for *rho* scRNA |
| *Kpn*Ⅰ-pTargetA-*rof* | GGGGTACCaaaactcctttcgaatcagggttttagagctagaaatag | for *rof* scRNA |
| For replacing promoter |  |  |
| TargetF-R | GGACTAGTATTATACCTAGGACTGAGCTAGCTGTCAAG | for TargetF |
| Target-purRp-F | GGACTAGTtggccgatcttgacgaaaagGTTTTAGAGCTAGAAATAG | for purRp sgRNA |
| Target-fruRp8-F | GGACTAGTgcggcaactgcttttattccGTTTTAGAGCTAGAAATAG | for fruRp8 sgRNA |
| Target-soxSp-F | GGACTAGTcagttcgttaattcatctgtGTTTTAGAGCTAGAAATAG | for soxSp sgRNA |
| Target-hdfRp-F | GGACTAGTactagctcggtcaaagaattGTTTTAGAGCTAGAAATAG | for hdfRp sgRNA |
| Target-treRp3-F | GGACTAGTattcagcaatgggtaaagtcGTTTTAGAGCTAGAAATAG | for treRp3 sgRNA |
| Target-cueRp6-F | GGACTAGTgcctcctgtgctttgttaaaGTTTTAGAGCTAGAAATAG | for cueRp6 sgRNA |
| purRp-up-dF-*EcoR*Ⅰ | CGGAATTCcattacgccaactgcataggcac | for the purRp substitution |
| purRp-up-dR | aaatgacgggagattttttcatcacagtgtg |  |
| purRp-P37-UTR-F | gaaaaaatctcccgtcatttCTTACATGAAAAAGGTTCTTGAC |  |
| purRp-P37-UTR-R | ATTTATTACCTCCTTAATTCCGAAATAGAAAAATGAC |  |
| purRp-dn-dF | GGAATTAAGGAGGTAATAAATattgaccccttcctgacgctcc |  |
| purRp-dn-dR-*Kpn*Ⅰ | GGGGTACCcatcaccagcagaccatcgacgc |  |
| fruRp8-up-dF-*EcoR*Ⅰ | CGGAATTCaatgaatcaggcgcgttatcccg | for the fruRp8 substitution |
| fruRp8-up-dR | tgacatgttgggctgtaaattgcgc |  |
| fruRp8-P37-UTR-F | caatttacagcccaacatgtcaCTTACATGAAAAAGGTTCTTG |  |
| fruRp8-P37-UTR-R | ATTTATTACCTCCTTAATTCCGAAATAGAAAAATGAC |  |
| fruRp8-dn-dF | GGAATTAAGGAGGTAATAAATtgaatttaacccataccagtac |  |
| fruRp8-dn-dR-*Kpn*Ⅰ | GGGGTACCagcgcgacaatcgggaacgggtc |  |
| soxSp-up-dF-*EcoR*Ⅰ | CGGAATTCgaccggaaaacaaactaaagcgc | for the soxSp substitution |
| soxSp-up-dR | atggaaaagaaattaccccgcattaaagcgc |  |
| soxSp-P37-UTR-F | tgcggggtaatttcttttccatCTTACATGAAAAAGGTTCTTG |  |
| soxSp-P37-UTR-R | ATTTATTACCTCCTTAATTCCGAAATAGAAAAATGAC |  |
| soxSp-dn-dF | GGAATTAAGGAGGTAATAAATactgaaaagaggcagatttatg |  |
| soxSp-dn-dR-*Kpn*Ⅰ | GGGGTACCctgaaggcgtcgaaactgaggag |  |
| hdfRp-up-dF-*EcoR*Ⅰ | CGGAATTCgttattgaaagttgcacgaaccattg | for the hdfRp substitution |
| hdfRp-up-dR | caggatggcggaaagctttacgacg |  |
| hdfRp-P37-UTR-F | cgtaaagctttccgccatcctgCTTACATGAAAAAGGTTCTTG |  |
| hdfRp-P37-UTR-R | ATTTATTACCTCCTTAATTCCGAAATAGAAAAATGAC |  |
| hdfRp-dn-dF | GGAATTAAGGAGGTAATAAATatcataacgacaaataattttgcgg |  |
| hdfRp-dn-dR-*Kpn*Ⅰ | GGGGTACCaattcgtccattttgggcgcttc |  |
| treRp3-up-dF-*EcoR*Ⅰ | CGGAATTCgcagattccacttccgcctg | for the treRp3 substitution |
| treRp3-up-dR | tgtcagcaggtattatatcgccatagatgctacg |  |
| treRp3-P37-UTR-F | ggcgatataatacctgctgacaCTTACATGAAAAAGGTTCTTG |  |
| treRp3-P37-UTR-R | ATTTATTACCTCCTTAATTCCGAAATAGAAAAATGAC |  |
| treRp3-dn-dF | GGAATTAAGGAGGTAATAAATcaggacaacaggatgcaaaatc |  |
| treRp3-dn-dR-*Kpn*Ⅰ | GGGGTACCattgccccttcgtcgtcataacag |  |
| cueRp6-up-dF-*EcoR*Ⅰ | CGGAATTCtcgtcgtcatcggcgctttactg | for the cueRp6 substitution |
| cueRp6-up-dR | gaccgccaccacaataatacaggaag |  |
| cueRp6-P37-UTR-F | ctgtattattgtggtggcggtcCTTACATGAAAAAGGTTCTTG |  |
| cueRp6-P37-UTR-R | ATTTATTACCTCCTTAATTCCGAAATAGAAAAATGAC |  |
| cueRp6-dn-dF | GGAATTAAGGAGGTAATAAATatgaacatcagcgatgtagc |  |
| cueRp6-dn-dR-*Kpn*Ⅰ | GGGGTACCtcagcgccagcgaggatctc |  |
| For qPCR |  |  |
| Q-*soxS*-F | gtggtacttgcaacgaatgttcc | qPCR for *soxS* |
| Q-*soxS*-R | gatatcaaaaatcggacgctcgg |  |
| Q-*hdfR*-F | gagctttcgaatcagacaactgg | qPCR for *hdfR* |
| Q-*hdfR*-R | ctcatgagcgtttctgcataagg |  |
| Q-*cra*-F | cgcatcgctaactatcttgaacg | qPCR for *cra* |
| Q-*cra*-R | catcaacctgacgctgtaaaagg |  |
| Q-*treR*-F | gagtgctgaaacggcgtaatatc | qPCR for *treR* |
| Q-*treR*-R | cgtcataacagaccgaagcaaag |  |
| Q-*purR*-F | gaacaatcttgagaaacagcggg | qPCR for *purR* |
| Q-*purR*-R | tatggcgatactcttccagcatc |  |
| Q-*cueR*-F | gaaaacggttatcgcacctacac | qPCR for *cueR* |
| Q-*cueR*-R | ggtcgttaaacagattcaccagc |  |
| Q-*cysG*-F | ttgctctgcctattcgggtattc | qPCR for *cysG* |
| Q-*cysG*-R | tcaaccccatatagaacaccagc |  |

**Table S2 Effect of the addition of trehalose on the production of** phenyllactic acid

| trehalose (g/L) | Host strain | Plasmid | OD_600_ | PhLA(g/L) |
| --- | --- | --- | --- | --- |
| 0 | PHE02(P_treR_::P37) | pZBK-P_esaR_-Cn*ldhA* | 8.84±0.19 | 5.32±0.05 |
| 0.01 |  |  | 9.63±0.07 | 5.39±0.03^n.s.^ |
| 0.1 |  |  | 9.53±0.10 | 5.48±0.01* |
| 0.5 |  |  | 9.66±0.08 | 5.59±0.01** |
| 1 |  |  | 9.22±0.13 | 5.75±0.03**** |
| 10 |  |  | 9.34±0.12 | 5.66±0.01**** |

**Table S3 Effect of the addition of CuSO_4_ on the production of** phenyllactic acid

| CuSO_4_(mM) | Host strain | Plasmid | OD_600_ | PhLA(g/L) |
| --- | --- | --- | --- | --- |
| 0 | PHE02(P_cueR_::P37) | pZBK-P_esaR_-Cn*ldhA* | 10.68±0.00 | 4.05±0.06 |
| 1 |  |  | 11.15±0.25 | 4.07±0.00^n.s.^ |
| 5 |  |  | 11.34±0.12 | 4.28±0.07^**^ |
| 10 |  |  | 10.36±0.05 | 4.10±0.00^n.s.^ |
| 100 |  |  | 9.21±0.08 | 3.29±0.03**** |
| 200 |  |  | 11.77±0.01 | 2.21±0.05**** |
| 500 |  |  | 10.24±0.09 | 1.41±0.03**** |

**Table S4 Production of aromatic chemicals in a 2 L shake flask**

| Strain | OD600 | Titer (g/L) | Yield (mg/g) |
| --- | --- | --- | --- |
| **phenyllactic acid** |  |  |  |
| PHE02 | 11.15±0.13 | 4.79±0.02 | 108.7±0. 5 |
| PHE02(P_cra_::P37) (pZBK-P_esaR_-Cn*ldhA*) | 10.29±0.12 | 5.31±0.00 | 120.4±0. 0 |
| PHE02(P_treR_::P37) (pZBK-P_esaR_-Cn*ldhA*) | 11.05±0.21 | 4.60±0.01 | 104.3±0. 3 |
| PHE02(P_soxS_::P37) (pZBK-P_esaR_-Cn*ldhA*) | 10.53±0.52 | 5.47±0.02 | 124.1±0. 4 |
| PHE02(P_cueR_::P37) (pZBK-P_esaR_-Cn*ldhA*) | 10.42±0.04 | 5.73±0.21 | 130.0±4.9 |
| **Caffeic acid** |  |  |  |
| TYR14B1 (pZEA-RgTAL-PaHpaB-SeHpaC) | 12.8±1.1 | 425.8±6.9 mg/L | 38.4±0.1 |
| TYR14B1 (P_fruR:_:P37) (pZEA-RgTAL-PaHpaB-SeHpaC) | 11.6±1.2 | 864.8±10.1 mg/L | 56.5±0. 3 |
| **Tyrosol** |  |  |  |
| TYR-14B1(pZEA(U)-SpyTag-aro10-linker-yahK) | 4.35±0.50 | 0.35±0.01 | 13.8±0.1 |
| TYR-14B1(P_purR:_:P37) (pZEA(U)-SpyTag-aro10-linker-yahK) | 5.21±0.05 | 2.43±0.01 | 60.7±0.0 |
| TYR-14B1(P_hdf:_:P37) (pZEA(U)-SpyTag-aro10-linker-yahK) | 5.49±0.18 | 2.22±0.03 | 56.2±0.0 |


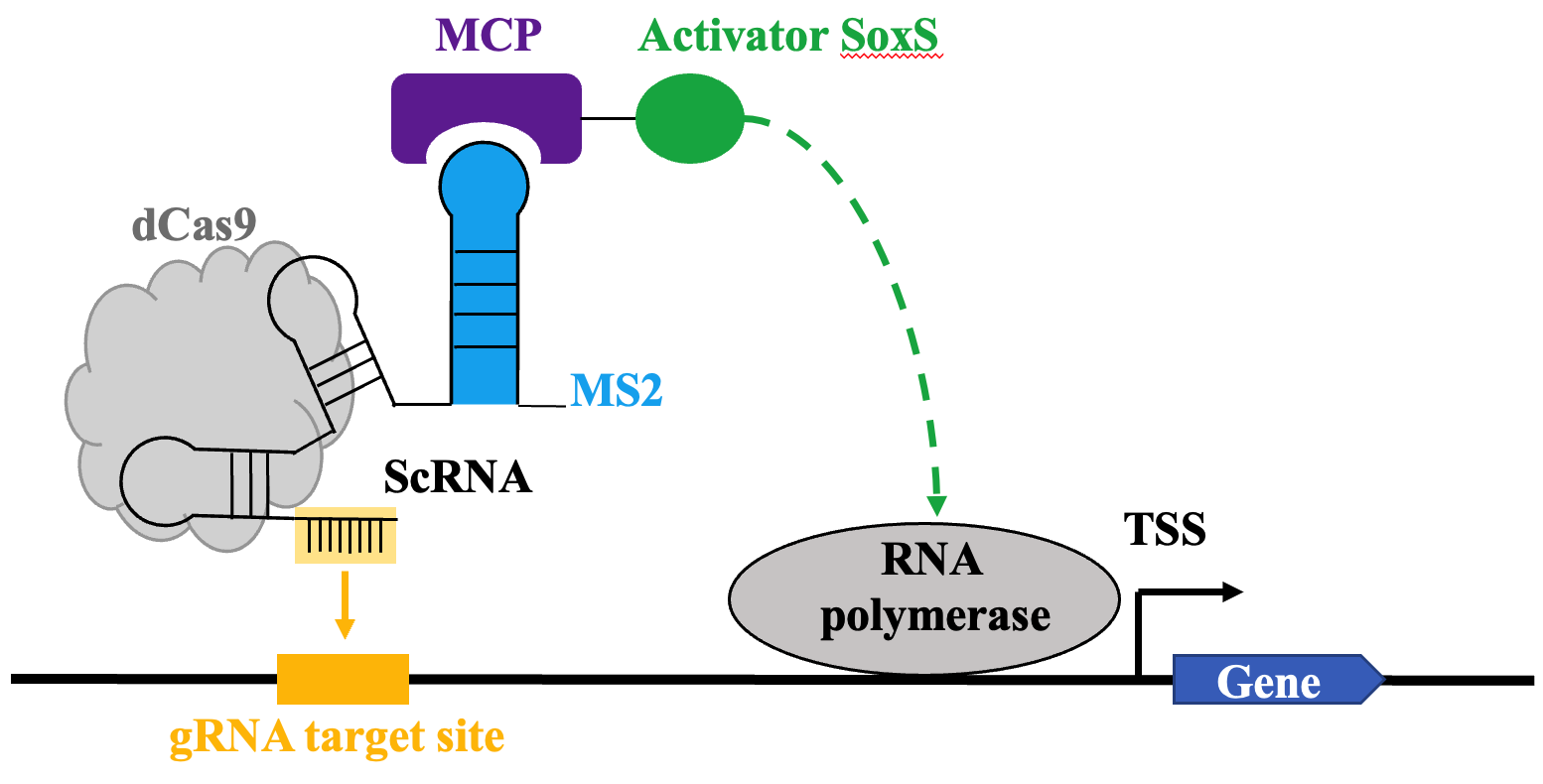


Fig. S1 CRISPR activation

(A)

(B)

(C)

Fig. S2 Tolerance of *E. coli* to phenyllactic acid (A), caffeic acid (B) and tyrosol (C). *E. coli* PHE02 for phenyllactic acid; *E. coli* TYR-14B1 for caffeic acid and tyrosol

Fig. S3 Effects of CRISPRa of the transcritional facors on growth of the phenyllactic acid producing *E. coli* PHE02 (pZBK-P_esaR_-Cn*ldhA*)

Phenyllactic acid Caffeic acid Tyrosol

Fig.S4 Structure of Phenyllactic acid, caffeic acid and tyrosol
